# A 30-year journey of trial and error towards a tolerogenic AIDS vaccine

**DOI:** 10.1007/s00705-018-3936-1

**Published:** 2018-07-24

**Authors:** Jean-Marie Andrieu, Wei Lu

**Affiliations:** Institut de Recherche sur les Vaccins et l’Immunothérapie des Cancers et du SIDA, Centre Universitaire des Saints Pères, Université de Paris-Descartes, 45 Rue des Saints Peres, 75006 Paris, France

## Abstract

Since 1985, we have tested several immunological approaches to suppressing HIV replication in HIV-infected patients and to prevent HIV acquisition in uninfected people. Here, after briefly reviewing our studies on immunosuppressive treatments and therapeutic dendritic cell-based therapies, we examine in more detail our work on the tolerogenic vaccines we developed against AIDS in Chinese macaques. The vaccine consisted of inactivated SIVmac239 particles adjuvanted with the Bacillus of Calmette and Guerin (BCG), *Lactobacillus plantarum* (LP), or *Lactobacillus rhamnosus* (LR). Without adjuvant, the vaccine administered by the intragastric route induced the usual simian immunodeficiency virus (SIV)-specific humoral immune responses but no post-challenge protection. In contrast, out of 24 macaques that were immunized with the adjuvanted vaccine and challenged intrarectally with SIVmac239 or SIVB670, 23 were sterilely protected for up to 5 years, while all control macaques were infected. On the other hand, all macaques of Indian origin that were immunized with the same adjuvanted vaccine were not protected. We then discovered that vaccinated Chinese macaques developed a previously unrecognized class of non-cytolytic MHC-Ib/E-restricted CD8^+^ T cells (or CD8^+^ T-Regs) that suppressed the activation of SIV RNA-infected CD4^+^ T cells and thereby inhibited the (activation-dependent) reverse transcription of the virus and prevented the establishment of SIV infection. Finally, we found a similar population of HLA-E-restricted CD8^+^ T-Regs in human elite controllers (a small group of HIV-infected patients whose viral replication is naturally inhibited). Ex vivo, their CD8^+^ T-Regs suppressed viral replication in the same manner as those of vaccinated Chinese macaques. It is noteworthy that all of these elite controllers had a homo- or heterozygous HLA-Bw4-80I genotype. Taking into account the longevity and the high percentage of vaccine-protected Chinese macaques together with the concomitant identification of a robust ex vivo correlate of protection and the discovery of similar CD8^+^ T-Regs in human elite controllers, preventive and therapeutic HIV vaccines should be envisaged in humans.

## Introduction

Human immunodeficiency virus (HIV) infection occurs mainly via the sexual and anal mucosa. It is now agreed that only the “transmitter/founder” HIV or simian immunodeficiency virus (SIV) virions that bear specific envelope “signatures” can cross the epithelial barrier of genital mucosa [1, 2]. Once the transmitter/founder virus has penetrated the mucosa, it makes contact with its target CD4^+^ T cell. Then, the virus attachment to CD4 receptors and CCR5 co-receptor is followed within two hours by the presentation at the plasma membrane of Gag and Pol antigens derived from incoming virions [3]. At the same time, viral RNA enters the target Gag/Pol^+^CD4^+^ T cell. Further development of the infection now depends on the activation status of the HIV-RNA-infected Gag/Pol^+^ CD4^+^ T cell. If it is in a quiescent/non-activated state, HIV RNA reverse transcription is very inefficient or abortive [4–6], and the infectious process is arrested. In contrast, if the HIV-RNA-infected, Gag/Pol^+^ CD4^+^ T cell is in an activated state, HIV RNA is readily reverse transcribed into HIV DNA. This step is then irreversibly followed by a cascade of cellular events ending in the release of large amounts of HIV virions that now replicate easily in the surrounding activated CD4^+^ T cells [7, 8]. Very quickly, infectious activated CD4^+^ T cells as well as free viral particles spread through the organism. Within a week, virus-specific cytotoxic T-lymphocytes (CTLs) are produced, followed by antiviral antibodies. However, viral envelope proteins have already mutated and thereby escaped antibody neutralization. HIV infection can thus perpetuate in a chronic manner. The same steps are observed with SIV in the rhesus macaque (RM) model.

Since 1983, when the virus was first discovered, most vaccinologists thought that, by using ongoing advances in immunological knowledge and the tools of modern molecular biology, they would finally find HIV vaccines that could stimulate the generation of antibodies capable of neutralizing the virus before it infects the first encountered CD4^+^ T cell, its principal target cell, and/or CTLs capable of destroying the first already infected CD4^+^ T cells at the mucosal border.

Until now, however, out of the large number of vaccine trials performed in RMs, only one (2013) was found to induce post-challenge sterile protection against SIV in 50% of vaccinated RMs [9]. Also in humans among more than hundred vaccine prototypes [10], the RV144 HIV trial (2009) is the only one to have produced a modest (32%) and short-term protection (3 years) against virus acquisition [11], while all of the other trials gave negative results.

Altogether, in spite of 30 years of research efforts, the picture of the HIV vaccine field is disappointing and demands new ideas and experimental work.

In our research, since 1985, we have imagined and tested several immunological approaches to trying to suppress HIV replication in infected patients and prevent HIV acquisition in uninfected people.

Here, after a brief overview of our experimental work on immunosuppressive treatments and therapeutic dendritic-cell-based therapies, we review in more detail our work on tolerogenic mucosal vaccines.

## Imunosuppressive drugs

In 1986, we pointed out that “*T4*-*cell activation (whether triggered by the virus, viral proteins, or by any other stimulus) was an absolute requirement for significant viral replication”* [12]. On the basis of this suspicion, which is now common knowledge [7, 8], we hypothesized that, by suppressing CD4^+^ T cell activation, it might be possible to suppress viral replication. On this basis, we tested the hypothesis that, by administering immunosuppressive drugs that are capable of fighting immune cell activation, such as cyclosporine and prednisolone, to infected patients viral replication could be potentially controlled. Although we showed that both of these drugs had a positive impact CD4^+^ T cell counts on HIV in infected patients, none of them were capable of suppressing HIV replication [13, 14].

## Dendritic-cell-based therapeutic vaccines

At the end of the nineties, after having abandoned the idea of suppressing viral replication through nonspecific systemic immune suppression, we turned our interest to virus-specific stimulation of dendritic cells (DCs) because we had shown that DCs loaded with chemically inactivated HIV-1 induced CTLs that suppressed viral replication in vitro by killing infected cells [15].

In 2003 and 2004, on this basis, we developed therapeutic vaccines made of ex vivo-prepared autologous DCs loaded with inactivated SIV or HIV. These vaccines administered subcutaneously to SIV-infected macaques and then to HIV-infected patients had a favorable impact on SIV and HIV replication [16, 17]. However, we abandoned the project of using DC-based vaccines because their preparation was expensive and incompatible with widespread use, and we imagined vaccines that would be able to act directly on DCs in vivo.

## The Bacillus of Calmette and Guerin, a tolerogenic adjuvant discovered by serendipity

We speculated that, instead of stimulating DCs by loading them ex vivo with inactivated virus, it might be more effective to develop a vaccine (made of the same iSIVmac239 particles) capable of acting directly in vivo on DCs. Because the only direct access to DCs is the mucosal route, our first vaccine prototype was administered intravaginally. Moreover, to increase mucosal DC stimulation, the vaccine was adjuvanted with the Bacillus of Calmette and Guerin (BCG), which has been shown to strongly stimulate mucosal DCs and Langerhans cells [18, 19].

In early 2006, seven Chinese RMs, which are now considered a better model than Indian RMs [20–24], were inoculated intravaginally with a vaccine comprising iSIV adjuvanted with BCG (strain SSI 1331). A booster immunization was administered two months later. Five control RMs were given BCG alone. At four months post-immunization, the 12 RMs were challenged by the intrarectal route with SIVmac239 (40,000 TCID_50_).

The results of this challenge were highly unexpected; four out of the seven vaccinated RMs became sterilely protected and had undetectable levels of plasma SIV RNA and cellular proviral DNA; the three remaining vaccinated RMs showed a mild SIV RNA peak followed by low viral set-points while the five control animals showed typical primary infection. However, in contrast to all established human and veterinary vaccines, none of the protected macaques developed anti-SIV antibodies [25].

In view of these unusual findings, we decided to test whether our vaccine would work in the same manner when administered by another mucosal route. We chose the intragastric route because, if successful, it could prefigure a human oral vaccine. The vaccine (adjuvanted with BCG) was thus administered through a gastric tube to four RMs; booster vaccinations were repeated at days 30 and 60; four control RMs were given BCG alone.

By day 90, an intrarectal challenge with SIVmac239 (100,000 TCID_50_) was given to the eight animals. The four control RMs showed typical primary infection. In strong contrast, all four vaccinated RMs were and remained sterilely protected, as indicated by the absence of any detectable SIV RNA and DNA in plasma and blood cells, suggesting that SIV infection was blocked at entry by mucosal immunity. SIV-specific antibodies and γ-interferon-releasing T-cell responses were undetectable and remained absent post-challenge.

We thus faced the unexpected picture of a mucosal vaccine made of iSIV adjuvanted with BCG that induced the suppression of SIV-specific humoral and cellular responses and at the same time induced sterile post-challenge protection.

Taking into account that SIV (or HIV)-specific CD4^+^ T-cell activation is the driving force of the initial burst of viral replication in vivo [26–28] and, conversely, that the suppression of CD4^+^ T-cell activation by local application of an anti-inflammatory agent has been shown to prevent mucosal SIV transmission [29], the only hypothesis that could reconcile these apparently contradictory observations was that our vaccine had induced SIV-specific tolerance, i.e., the suppression of SIV-specific CD4^+^ T-cell activation, which in turn had prevented viral reverse transcription from occurring [4–8].

### *Lactobacillus plantarum* as a tolerogenic adjuvant

In 2009, we decided, in place of BCG, to test another bacterial adjuvant, *Lactobacillus plantarum* (LP), because this intestinal commensal bacterium had been suspected to induce some forms of tolerance [30–32]. We therefore immunized a group of 16 RMs via the intragastric route; eight received iSIV adjuvanted with LP, four received LP only, and four received iSIV only [33]. Again, pre-challenge SIV-specific antibody and γ-interferon-producing cell responses were absent in the eight vaccinated RMs, while they were present in the four RMs immunized with iSIV only. By day 90 post-immunization, all 16 animals were challenged intrarectally with SIVmac239. Once more, the eight RMs immunized with iSIV^+^ LP were sterilely protected without any SIV-specific humoral and cellular responses, while the eight control animals were infected. Sterile protection was fully confirmed after a second intrarectal challenge performed two months later in four of the eight already protected RMs. The same four animals again remained protected against a third intrarectal challenge performed eight months later with 100,000 TCID_50_ of the antigenically distinct SIVB670 strain. This suggested that the vaccine was cross-protective, presumably through preventing the activation of CD4^+^ T cells infected by another SIV strain.

## Long-term protection of vaccinated macaques validated by an external research group

To study the longevity of the protection, in March 2010, we administered the iSIV/LP vaccine intragastrically to eight new RMs, while four received iSIV only, and the remaining four received LP only [25]. A first intrarectal challenge was administered 13 months after vaccination, and we found that one vaccinated RM was infected; the seven others were again protected, as indicated by the absence of the emergence of any SIV RNA and DNA in plasma or blood cells, or in rectal mucosa and pelvic lymph node lymphocytes. Pre- (and post-) challenge SIV-specific humoral and cellular responses were also suppressed in these seven RMs, whereas the four RMs immunized with iSIV alone developed the usual plasma SIV-specific IgM and IgG antibodies and cellular responses. Twenty-two months later, we decided to administer a new intrarectal challenge to the seven protected RMs to examine the longevity of their protection. However, our previous results were so unusual that we considered that validation by an external research group would be advisable. For that purpose, we asked Gianfranco Pancino, head of an AIDS research group from the Pasteur Institute, to perform this late intrarectal challenge. This SIV mac239 (100,000 TCID_50_) challenge was administered in February 2013 in the seven already-protected RMs and four new controls. Four weeks later, full confirmation was brought that the seven RMs remained sterilely protected 35 months post-vaccination, while the four control RMs became infected.

## Absence of immune reactivity of Indian macaques to intra-gastric immunization

Following the suggestion of Jose Esparza, then adviser for AIDS research at the Bill and Melinda Gates Foundation, Guido Silvestri (Atlanta) agreed to replicate our study, not in Chinese RMs, but in Indian RMs, the most widely used model in the United States. Inactivated SIVmac239 and LP were transferred from our labs to Atlanta, and both their quality and their amount were tested by specialists before use.

In March 2015, 17 Indian RMs received iSIV plus LP intragastrically, ten received iSIV only, another ten received LP only, and 17 Indian RMs received a sham intragastric immunization. Intrarectal challenge with SIV mac239 performed three months later resulted in rapid infection in all groups of immunized Indian RMs and unvaccinated controls [34]. Importantly, however, the vaccine made of iSIV particles given alone to 10 Indian RMs did not induce any SIV-specific antibody response (the cellular response was not checked). In contrast, the same iSIV particles administrated alone to 10 Chinese RMs via the same intragastric route were immunogenic, as shown by the SIV-specific humoral and cellular immune responses they generated. As a consequence of this absence of digestive immune reactivity, the 17 Indian RMs vaccinated with iSIV^+^ LP and challenged intrarectally, not surprisingly, were infected. The absence of immunoreactivity of Indian RMs toward the ingested vaccine (iSIV particles) could have resulted from the destruction or the damage of these particles before or after their intragastric administration; a difference in the genetic background of the two RM subspecies could also be envisaged [22, 23].

### *Lactobacillus rhamnosus* as a tolerogenic adjuvant

Because the *Lactobacillus plantarum* strain ATCC8014 used to vaccinate RMs had never been used in humans, we decided to test the commercial probiotic strain *Lactobacillus rhamnosus* (LR) (LCR35, Biose, 15,000 Aurillac, France) as a bacterial adjuvant. Four RMs were vaccinated intragastrically with iSIV and LR; two control RMs received LR only, and two iSIV received only [25].

Twelve weeks post-vaccination, the eight RMs were administered SIVmac239 intrarectally. The four vaccinated RMs were sterilely protected, while the four control RMs were infected.

## Suppression of SIV-specific CD4^+^ T-cell activation and viral replication by CD8^+^ T-Regs

A central question was how a mucosal vaccine made of iSIV particles adjuvanted with BCG, LP or LR induced, both SIV-specific tolerance (defined by the absence of SIV-specific CD4^+^ T-cell activation and antibody production) and a post-challenge sterile protection in Chinese RMs, while the same vaccine administered without adjuvant induced a classical immune response and no post-challenge protection.

Several ex vivo experiments allowed us to demonstrate that the suppression of SIV-specific CD4^+^ T-cell activation was induced by a previously unrecognized class of non-classical MHC-Ib/E-restricted CD8^+^ T cells, which we named CD8^+^ T-Regs.

In spite of our efforts, we did not succeed in defining the phenotype of these CD8^+^ T-Regs on thawed samples. However, we performed experiments demonstrating their functional properties: SIV-specific CD8^+^ T-Regs from vaccinated RMs prevented SIV replication in autologous or allogenic infected CD4^+^ T-cells, but in a MHC-Ib/E-restricted manner. This antiviral activity of CD8^+^ T-Regs was shown to require cell-to-cell contact; moreover, CD8^+^ T-Regs did not induce any CD4^+^ T-cell cytotoxicity.

We showed that CD8^+^ T-Regs had no antiviral activity on already activated CD4^+^ T cells. Finally, we verified the absence of a role of classical regulatory T cells (CD25^+^FoxP3^+^CD4^+^ T cells) in the suppression of CD4^+^ T cell activation and viral replication.

Overall, vaccine-induced non-classical MHC-Ib/E-restricted CD8^+^ T-Regs prevented (by contact) the activation of SIV-RNA-infected CD4^+^ T cells, which in turn prevented the (activation-dependent) reverse transcription of SIV RNA into SIV DNA and the subsequent cascade of events ending in virus replication and release.

To measure the antiviral activity of the vaccine, we established a virus suppression assay where we measured SIV concentrations in supernatants of infected CD4^+^ T cells (from healthy RMs) cultivated for 5 days in the presence or absence of fresh CD8^+^ T cells collected at several time points before and after vaccination of RMs.

**Fig. 1:**
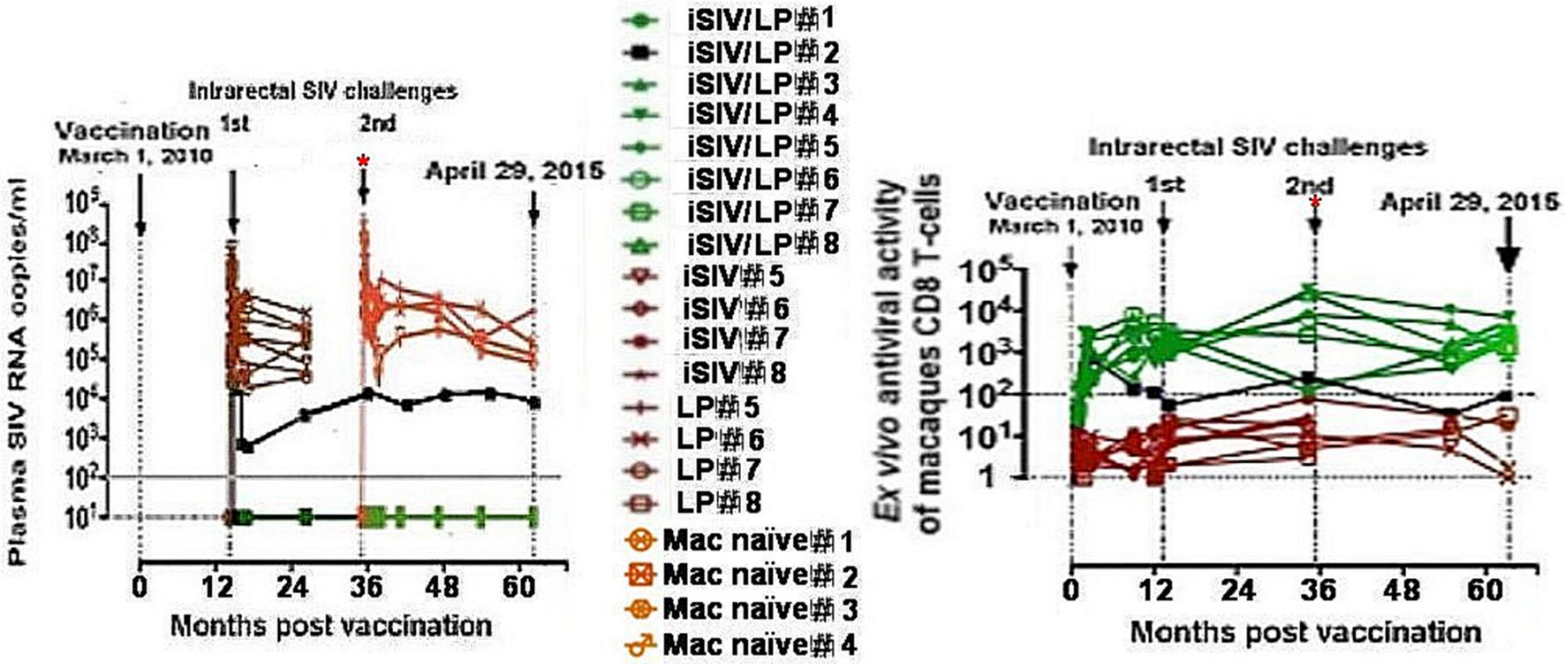
Plasma SIV RNA loads (left) and ex vivo antiviral activity (right) in eight macaques that were immunized intragastrically with iSIV adjuvanted with LP (7 in green, 1 in black), in eight macaques immunized with iSIV only or LP only (red), and in four naive macaques (orange) after two intrarectal challenges performed 13 and 35 months post-immunization. One vaccinated macaque was not protected after the first challenge (black); the second challenge (red asterix) was performed under the supervision of GF Pancino and his group from the Pasteur Institute) (color figure online)

In our second series of eight vaccinated RMs, seven were protected (Fig. 1, left, green line); all seven showed a strong pre-challenge CD8^+^ T-Reg-mediated antiviral activity that remained stable until 5 years post-vaccination (Fig. 1, right, green lines). In contrast, the antiviral activity of CD8^+^ T-Regs from the only animal that was later shown not to be protected (Fig. 1, left, black line) increased until month 3 but then decreased until reaching a baseline level similar to those of unprotected control RMs (Fig. 1, right, black line). Altogether, this suggested that post-vaccination measures of CD8^+^ T-Reg-mediated antiviral activity represented a pathogenic correlate of protection ex vivo.

## Similar CD8^+^ T-Regs in HIV-infected elite controllers and vaccinated Chinese macaques

Having discovered in vaccinated Chinese RMs that the suppression of viral replication resulted from vaccine-induced CD8^+^ T-Regs, the question was then whether similar CD8^+^ T-Regs could exist in human “elite controllers”, a small percentage of HIV-infected patients whose viral replication is naturally inhibited for long periods of time.

Ten elite controllers were studied [35]. In nine of them, we found HIV-specific non-cytolytic HLA-E-restricted CD8^+^ T-Regs that displayed the same in vitro suppression mechanisms as those discovered in vaccinated RMs. These nine HIV-infected patients had the same homo-or heterozygous HLA-Bw4-80I genotype. As shown in ex vivo experiments, by inhibiting the activation of HIV-infected CD4^+^ T cells, CD8^+^ T-Regs of elite controllers maintained HIV replication suppression. Our discovery of this CD8^+^ T-Reg population in human elite controllers provides a further input for the design of a tolerogenic vaccine against AIDS in humans.

## CD8^+^ T-Regs in the mouse model

CD8^+^ T-Regs had never been described previously in humans or RMs. However, although they are not cytolytic, our vaccine-induced CD8^+^ T-Regs resemble CD8^+^ T suppressor cells that targeted and eliminated abnormally activated antigen-specific CD4^+^ T cells in the mouse model, where the inhibitory interaction depends on recognition of surface Qa-1 (corresponding to MHC-IB/E in RMs and to HLA-E in humans) expressed by apparently activated target cells [36–38].

## Application to humans

Taking into account the longevity and the high percentage of protection induced by our intra-gastric vaccine adjuvanted by BCG, LP or LR (23/24 RMs), together with the identification of a strong pathogenic correlate of protection ex vivo and the discovery of similar CD8^+^ T-Regs in human elite controllers, preventive and therapeutic HIV vaccinations should be envisaged in humans.

The safe manufacture of a human vaccine made of inactivated HIV particles adjuvanted with LR requires adequate GMP technology and strong controls.

Once an HIV vaccine has been safely manufactured and approved by regulatory agencies and ethical bodies, the first phase 1 clinical trial should establish whether vaccinated HIV-infected patients with undetectable plasma viral load under antiviral treatment developed CD8^+^ T-Regs. This will be shown by measuring the antiviral activity of fresh CD8^+^ T-cells taken from patients before vaccination and at several time points after vaccination.

The results of such preliminary trials will provide the information required for considering whether further preventive and therapeutic efficacy trials should then be undertaken.

## Conclusions

Altogether, we started with the discovery by serendipity of an effective mucosal vaccine made of iSIV particles adjuvanted with BCG in the Chinese RM model. We then found that the vaccine worked in the same manner when adjuvanted with LP or LR. We showed further that the adjuvanted vaccine resulted in the development of a population of CD8^+^ T-Regs that had the ability to prevent the activation of SIV-RNA-infected CD4^+^ T cells and in turn to prevent the (activation-dependent) reverse transcription of SIV RNA into SIV DNA and the subsequent cascade of events ending in virus replication and release.

Importantly, we also demonstrated that the same type of CD8^+^ T-Regs exists in HIV-infected elite controllers with a particular HLA genotype.

Given that SIV and HIV require the same activated immune cells in which to replicate, the specific prevention from activation of SIV RNA CD4^+^ T cells by a tolerogenic vaccine approach offers an exciting new avenue in preventive and therapeutic HIV vaccine research.
